# Potential of an Exploitation of Acid-Tolerant Antimicrobial Microorganisms Evolving Enzyme Systems for the Utilization of Dairy By-products and Lignocellulosic Biomass to Lactic Acid

**DOI:** 10.3389/fbioe.2016.00092

**Published:** 2016-11-28

**Authors:** Grazina Juodeikiene, Daiva Zadeike, Elena Bartkiene, Vadims Bartkevics, Alexander Dikiy, Elena Shumilina

**Affiliations:** ^1^Food Science and Technology, Kaunas University of Technology, Kaunas, Lithuania; ^2^Food Safety and Quality, Lithuanian University of Health Sciences, Kaunas, Lithuania; ^3^Institute of Food Safety, Animal Health and Environment, Riga, Latvia; ^4^Department of Chemistry, University of Latvia, Riga, Latvia; ^5^Norwegian University of Science and Technology, Trondheim, Norway

**Keywords:** cheese whey, lignocellulosic substrate, lactic acid bacteria, enzyme activities, lactic acid production, fermentation process

## Introduction

Bioproduction of optically pure lactic acid (LA) has roused interest in the recent years due to its potential application in a wide range of fields, and there is a significant interest to further development of sustainable and cost-effective process. However, the efficient utilization of agro-industrial wastes for LA production still causes considerable challenges. The biotechnological LA production within the targeted cost still required the development of high-performance LA-producing microorganisms and the lowering of the costs of raw materials and fermentation process. Cheap biomass, such as starchy and cellulosic agricultural residues or by-products from the food industry, has a potential for the cost-effective production of LA, but raw materials also should have a high production rate and yield without by-product formation and the ability to be fermented with low pretreatment (Wee et al., 2006). Whereas the LA made by fermentation route refers optically active, consequently a suitable microorganism could selectively produce dextro (levo)-rotation enantiomers, and the greatest demand is for the l-LA isomer (Sheldon, 2011).

Targeted conversion of starchy substrates to LA can be performed using the amylolytic microorganisms (Gonzalez et al., 2007). Fungi species from *Rhizopus*, such as *Rhizopus oryzae* and *Rhizopus arrhizus*, excreate amylolytic activity that enables to convert starch directly into l-LA in the presence of oxygen (Hofvendahl and Hahn-Hägerdal, 2000). However, LA-producing microorganisms, including the fungus *R. oryzae*, have low productivity depended on the low reaction rate caused by mass transfer limitation (Okano et al., 2010). Most of the world’s commercial l-LA is produced by the fermentation of carbohydrates using homolactic microbes such as a variety of modified or developed strains of the genus *Lactobacilli* (Naveena et al., 2005; Ohkouchi and Inoue, 2006). This can be considered to be an advantage, since the productivity of the industrial process may become independent from oxygen supply.

Nowadays, the development of sustainable processes requires the efficient exploitation of food processing residues and maximization of the value derived from such waste source. In this field, the dairy industry by-products (e.g., whey, whey permeate) received considerable attention as a suitable carbon source, since they are substrates that do not require extensive purification, and can be the most appropriate option for the production of biodegradable polymers as well as for many other applications. While the efficient lactose consumption depends on the different factors affecting the LAB growth in the fermentation medium, the selection of LAB strain without complex nutritional requirements and pH regulation is one of the most important factors influencing LA production rate (Guha et al., 2013).

Our recent studies, related to the development of a cost-effective and sustainable process, focus on an application of novel acid tolerant microorganisms evolving different enzyme activities on the efficient LA stereoisomers production from different cereal based food residues and dairy by-products, including enzymatic hydrolysis and acid neutralization (Juodeikiene et al., 2016a,b).

## Bioconversion of Agro-Industrial and Dairy By-Products to Lactic Acid Using Antimicrobial LAB Strains

The application of microbial strains remaining genetically stable under processing conditions is the complete technological challenge of LA production. At the same time, industrial microorganisms should resist the competitive microbial contamination occurred in the raw material that represents a high risk for fermentation process. The newly isolated bacteriocins producing LAB indicating a wide antimicrobial spectrum (Digaitiene et al., 2012; Cizeikiene et al., 2013) could be the advantage. Furthermore, the development of LAB strains, able to produce the required enzyme activities in the natural substrate, is yielding the new applications and holds a great potential for fermentative LA production (Juodeikiene et al., 2016a,b). The effective biomass saccharification is an essential subject of the bioprocesses intensification. Studies on the adaptation of different lactobacilli and pediococci to different substrates, evaluated by sugar consumption rate and enzyme activities, show the considerably higher growth performance of tested strains in the lignocellulose-based medium (Juodeikiene et al., 2016a) as compared to dairy by-products (Juodeikiene et al., 2016b). The optically pure l-LA could be synthesized in a selected medium using a particular bacterial strain (Juodeikiene et al., 2016a,b).

## Intensification of a Fermentation Process for Production of LA

The application of commercial enzymes and LAB evolving required enzyme activities indicates the following tendency in the perspective of the biomass enzymatic pretreatment and fermentative LA production (Juodeikiene et al., 2016a,b).

As was confirmed by an extensive monosaccharide target analysis, the 15-fold increase in maltose, fructose, and glucose concentrations can be fixed after the wheat bran (WB) polysaccharide enzymatic pretreatment by commercial hemicellulase. Thus, the increase up to 60, 38, and 29% in consumption of glucose, xylose, and arabinose, respectively, could be reached during fermentation by LAB (Juodeikiene et al., 2016a). The maximum l-LA concentration and yield of 86 g kg^−1^ and 1.45 g g^−1^, respectively, could be achieved after the bioconversion of WB using enzymatic hydrolysis in combination with 48 h fermentation by *Pediococcus pentosaceus* KTU05-9 strain (Juodeikiene et al., 2016a). The whey pretreatment with commercial β-galactosidase could increase the lactose hydrolysis up to 36%, thereby resulting the l-LA production up to 28 g L^−1^ using *Pediococcus acidilactici* KTU05-7 strain for the 48-h fermentation (Juodeikiene et al., 2016b). With reference to the results, lactose consumption could reach the same level approximately at 15th- and 16th-h in hydrolyzed whey as compared to non-treated samples; therefore, lactose hydrolysis prior to the lacto-fermentation could reduce the fermentation time.

In the literature, WB pretreatment using acid hydrolysis (80°C; 20 h) showed a better performance than that without treatment, especially for l-LA yield (0.99 g g^−1^) (Li et al., 2010). Maximum l-LA concentration (33 g L^−1^) was reached employing barley bran hydrolyzates after dilute acid hydrolysis (Moldes et al., 2006). Also, the l-LA content of 9 g L^−1^ was obtained from soybean straw enzymatic hydrolyzate at 30°C when using *Lactobacillus casei* for the 54-h fermentation (Wang et al., 2015).

The tested antimicrobial LAB strains could be selected by excreted specific enzymatic activity dependent on the composition of fermentation medium and fermentation time. The fermentation of whey with the selected LAB might affect the saccharification process close to the one displayed within the applied commercial β-glucosidase (Juodeikiene et al., 2016b). Moreover, according to the Matijević et al. (2011), hydrolysis of whey lactose allows to reduce the fermentation time and increase the viable cell count of bacteria. The higher level of lactose hydrolysis initiates the higher LA productivity; however, the faster pH-value change in the hydrolyzed whey could cause the lower LAB β-galactosidase activity (Wang and Sakakibara, 1997). While the LAB producing bacteriocins are not sensitive for enzymatic treatment used for saccharafication of substrate (Narbutaite et al., 2008), it confirms the outlook of the LAB application for different bioprocesses including the saccharification of fermentation medium.

## The Advantage of the Use of Acid Tolerant LAB Strains for the Increasing the Efficiency of LA Production

A constant important objective during industrial production of LA is the cost reduction not only searching for cheap raw materials but also using of low-cost fermentation processes. Acid neutralization with traditional reagent CaCO_3_ at amounts between 10 and 25% (w/v) usually is applied in industry, mainly to make the processing easier and cheaper (Huang et al., 2005; Nakano et al., 2012). On the other hand, the LA extraction process is complicated because of the resulting high salt content, thus low pH reduces significantly the consumption of neutralizing agent in the fermentation stage and subsequent formation of gypsum in the product recovery stage. Microorganisms that have the capability to ferment raw materials rapidly and provide a high yield of required stereospecific LA under low pH and high temperature conditions are industrially desirable.

Currently, research on LA production from cheese whey is being conducted on the reduction of hazardous waste, because for microbial fermentation, less sensitive and low pH LAB strains could be applied. Experiment showed the different tolerance of tested strains to acidic medium that required the different contents of neutralizing reagent (Juodeikiene et al., 2016b). However, it should be indicated that amounts of neutralizing reagent were relatively low (2% w/v). The major advantage within the present investigations is the possibility to increase the effectiveness of fermentative LA production from whey reducing the costs of enzyme in the hydrolysis step, and neutralizing reagent in the fermentation step using acid tolerant LA bacteria strains with high β-galactosidase activity in all cases.

## Conclusion

Microorganisms, isolated from acidic spontaneous fermented cereal media, should be taken into consideration as starter cultures for agro-industrial by-products as well as dairy waste bioconversion to the LA. They demonstrate a longer microbial viability during the fermentation and also allow obtaining the higher concentrations of LA and the maximum productivity. Regular important objective of industrial LA production is the reduction of the cost of neutralizing reagent and avoid environmental problems encountered as a result of the gypsum formation. However, there is still a big need to produce the LA biotechnologically at the lowest costs not only by using cheap raw materials but also by improving the biomass pretreatment process to increase the availability of sugars by microorganisms. To accomplish this task, very promising achievements could be reached by evaluating the enzymatic activities excreting by particular LAB strain in the fermentation medium and based on the obtained results adjust the deficient activities of commercial enzymes. Economical evaluation of costs of the fermentative LA production showed that the operational costs that consist of raw materials, fermentation, electrodialysis, and hydrolysis costs contribute about 77% of the total costs (Juodeikiene et al., 2015). Thus, the combination of enzymatic pretreatment with selected acid tolerant antimicrobial organisms evolving enzyme systems appears to be promising for increasing the economical efficiency of fermentative LA production.

## Author Contributions

GJ was working on the concept of the efficiency evaluation of the LA production from dairy and cereal industry by-products. EB was working on optimization of LA production from cheese whey by using selected microorganisms. DZ was working on optimization of agro-industrial waste utilization to LA by using enzyme active microorganisms and their enzyme activity evaluation. AD and ES carried out the LA analysis.

## Conflict of Interest Statement

The authors declare that the research was conducted in the absence of any commercial or financial relationships that could be construed as a potential conflict of interest. The reviewer LR and handling editor declared their shared affiliation, and the handling editor states that the process nevertheless met the standards of a fair and objective review.
